# Supplementary material for: Perceived Help-Seeking Difficulty, Barriers, Delay, and Burden in Carers of People with Suspected Dementia
Source: Int J Environ Res Public Health. 2021 Mar 13;18(6):2956. doi: 10.3390/ijerph18062956 (PMC7999253; doi:10.3390/ijerph18062956)
Supplement: Supplementary file 1 [file ijerph-18-02956-s001.pdf]

International Journal of
Environmental Research and Public Health

MDPI

Supplementary Table. S1 Correlation matrix of key variables.

	1	2	3	4	5	6	7	8	9	10	11
**1. Age (person with dementia)**											
**2. Age (carer)**	0.27**										
**3. Education (carer)**	−0.10	−0.54**									
**4. Monthly income (carer)**	0.05	−0.34**	0.23								
**5. Time to help-seeking**	0.06	0.09	0.03	-0.00							
**6. Perceived difficulty**	−0.00	−0.06	−0.02	−0.00	0.06						
**7. ADCS-ADL**	−0.22*	−0.02	0.17	−0.17	−0.09	−0.25*					
**8. NPI-Q severity**	−0.03	−0.09	−0.02	0.19	0.36**	0.20*	−0.25**				
**9. Role strain**	0.00	−0.27**	0.12	0.09	0.16	0.18	−0.35**	0.59**			
**10. Self-criticism**	−0.03	−0.26**	0.10	0.06	0.08	0.41**	−0.16	0.20*	0.35**		
**11. Negative emotions**	0.02	−0.23*	0.07	0.06	0.16	0.23*	−0.38**	0.56**	0.76**	0.33**	

*$p<0.05$; **$p<0.01$

ADCS-ADL = Alzheimer's Disease Cooperative Study-Activities of Daily Living; NPI-Q = Neuropsychiatric Inventory-Questionnaire
